# New data on the metabolism of chloromethylisothiazolinone and methylisothiazolinone in human volunteers after oral dosage: excretion kinetics of a urinary mercapturic acid metabolite (“M-12”)

**DOI:** 10.1007/s00204-021-03100-5

**Published:** 2021-06-21

**Authors:** Thomas Schettgen, J. Bertram, T. Kraus

**Affiliations:** Institute for Occupational, Social and Environmental Medicine, Medical Faculty, RWTH Aachen University, Pauwelsstrasse 30, 52074 Aachen, Germany

**Keywords:** Biocide, Human biomonitoring, Mercapturate, Toxicokinetics

## Abstract

Methylisothiazolinone (MI) as well as the mixture of chloromethylisothiazolinone/methylisothiazolinone [MCI/MI (3:1)] are biocides that are used in a variety of products of every-day life. Due to the skin sensitizing properties of these biocides, their use has come under scrutiny. We have previously examined the human metabolism of MI and MCI after oral dosage of isotope-labelled analogues in human volunteers and confirmed *N*-methylmalonamic acid to be a major, but presumably unspecific human urinary metabolite. In the present study, we have investigated the urinary kinetics of a mercapturic acid metabolite of MI and MCI using the same set of samples. Four human volunteers received 2 mg of isotopically labelled MI and MCI separately and at least 2 weeks apart. Consecutive urine samples were collected over 48 h and were examined for the content of the (labelled) 3-mercapturic acid conjugate of 3-thiomethyl-*N*-methyl-propionamide (“M-12”), a known metabolite in rats. On a molar basis, M-12 represented 7.1% (3.0–10.1%) of the dose excreted in urine after dosage of MI. Excretion of this mercapturate was fast with a mean half-life of 3.6 h. Surprisingly, for MCI the mercapturate M-12 represented only 0.13% of the dose excreted in urine. Thus, this biomarker is highly specific for exposures to MI and might be used to distinguish between different exposure patterns of these biocides [use of MI or MCI/MI (3:1)] in the general population.

**Supplementary Information:**

The online version contains supplementary material available at 10.1007/s00204-021-03100-5.

## Introduction

Methylisothiazolinone (MI) and the mixture of chloromethylisothiazolinone/methylisothiazolinone [MCI/MI (3:1)] are biocides that are used for preservation of cosmetic products, water-based paints, cleaning agents, wet wipes or cooling lubricants in occupational settings (Scientific Committee of Consumer Safety 2009, 2016). These biocides can both be taken up via skin contact by cosmetics or wet wipes as well as inhalative, e.g. after application of cleaning agents or paints indoors (Lundov et al. 2014). In animal experiments, MI as well as MCI/MI (3:1) showed low chronic (NOAEL 66–94 mg/kg/day in rat 3-month oral toxicity) and developmental toxicity (NOAEL 30 mg/kg/day in rabbit developmental toxicity) with mainly local irritating effects at the point of entry. There were also no reports of genotoxic or carcinogenic effects (Burnett et al. 2010). Some in vitro-experiments point to a neurotoxic action of MI (Du et al. 2002; He et al. 2006). However, the toxicological evaluation of MI and MCI/MI (3:1) is dominated by their sensitizing potential. Both compounds are known skin allergens, so that their use in cosmetic products is regulated by law.

Until 2005, only use of MCI/MI (3:1) has been allowed in cosmetic products up to a maximum content of 15 ppm. In 2005, use of MI has been permitted as a standalone preservative in cosmetics up to a maximum content of 100 ppm (Regulation (EC) No. 1223/2009). In the following years, dermatologists have reported an increase in skin sensitizations against MI and MCI/MI (3:1) in the general population in various countries (Schwensen et al. 2017). This led to stronger restrictions in the cosmetic use of MI and MCI/MI (3:1), which finally ended up in a ban of both compounds in leave-on cosmetics (Regulation (EC) No. 1003/2014; Regulation (EC) No. 2016/1198) and a maximum content of 15 ppm in rinse-off cosmetics for both MI or MCI/MI (3:1) (Regulation (EC) No. 2017/1224).

However, the content of MI and MCI/MI (3:1) in household products remained untouched by these regulations (Garcia-Hidalgo et al. 2017). Thus, an exposure of the general population both to MI as well as MCI/MI (3:1) remains very likely. Besides the skin sensitizing properties, also inhalative exposure to these compounds has come under scrutiny. The (former) use of MCI/MI (3:1) and MI as humidifier disinfectant in South Korea was suspected to be the cause for the development of idiopathic lung injury in exposed persons (Park et al. 2020; Song et al. 2020).

In the search for a biomarker of exposure to MI and MCI, we have developed a method for the quantification of *N*-methylmalonamic acid (NMMA) as the main urinary metabolite of MCI and MI in animal experiments (Schettgen et al. 2017) and investigated excretion kinetics of NMMA after oral dosage of isotopically labelled MI and MCI in a human volunteer experiment (Schettgen and Kraus 2017). In various applications of our analytical method a background excretion of NMMA in urine of the general population over the last 20 years was confirmed with estimated median daily exposures of 0.35 µg/kg/bw for MI or 0.64 µg/kg bw for MCI/MI (3:1) for adults (Schettgen et al. 2020; Murawski et al. 2020). However, as NMMA is a common metabolite of both MI and MCI, our previous investigations did not allow to distinguish between exposures to MI and/or MCI/MI (3:1), adding substantial uncertainty to the dose extrapolations performed for the general population. As MCI shows higher reactivity towards skin and a higher sensitizing potency compared to MI (Berthet et al. 2017), a differential dose assessment is of interest for public health.

**Fig. 1:**
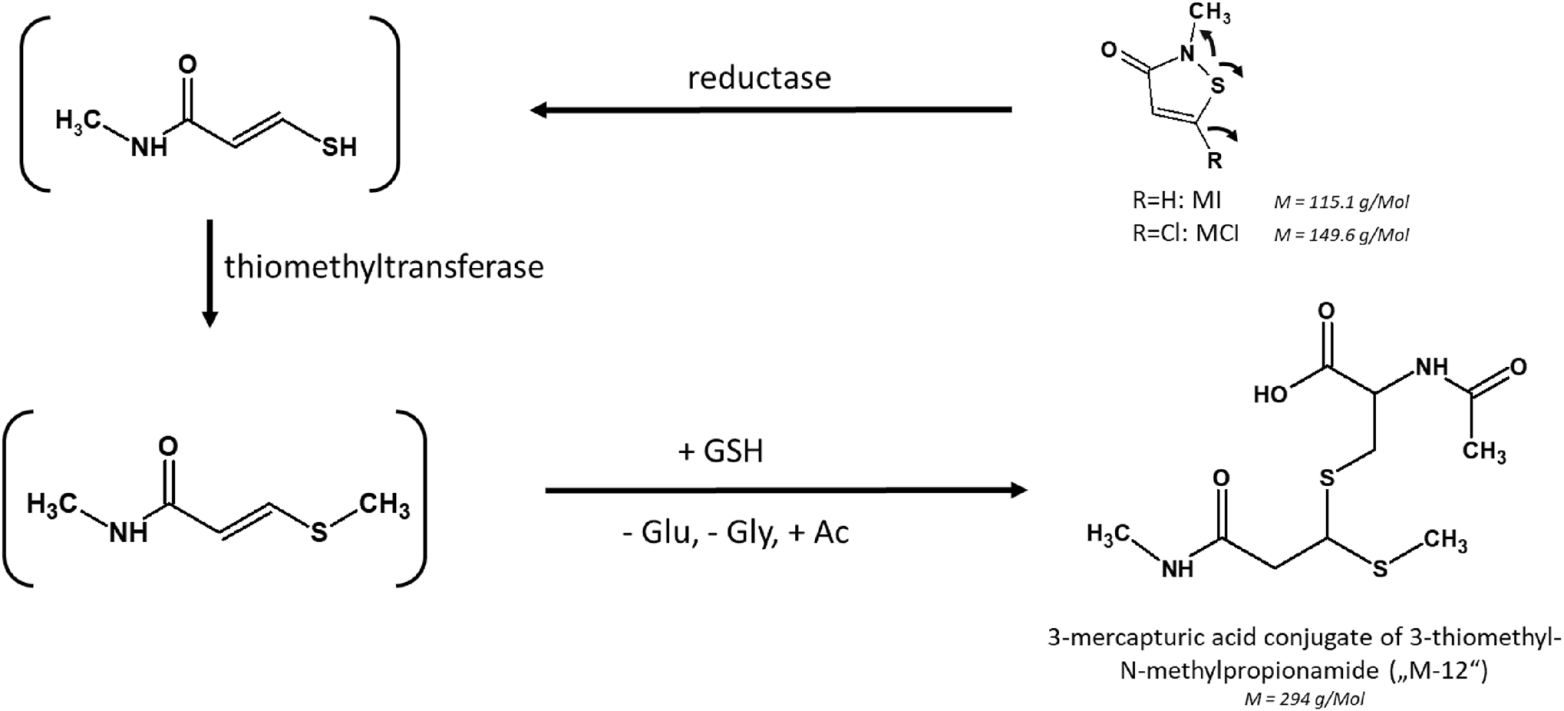
Simplified metabolism of MI and MCI, leading to the mercapturate M-12

To confirm our previous investigations and to further explore human metabolism of MI and MCI, we decided to develop an analytical method for an urinary mercapturate metabolite of MI and MCI, namely the 3-mercapturic acid conjugate of 3-thiomethyl-*N*-methyl-propionamide ((Acetylamino){[3-(methylamino)-1-(methylthio)-3-oxopropyl]thio}acetic acid or shortly “M-12”) and also found a background excretion for this biomarker at considerably lower levels than for NMMA (Schettgen et al. 2021). This metabolite has been reported to be excreted in urine between 10 and 23% of the administered dose within 24 h after oral dosage of 5 and 50 mg/kg bw of ^14^C-labelled MI to Sprague–Dawley rats (Burnett et al. 2010). A simplified metabolic scheme of MI, depicting the structures and formation of M-12 as biomarker is shown in Fig. 1.

As reported previously (Schettgen and Kraus 2017), we have orally dosed isotopically labelled isothiazolinones (^13^C_3_-MI and D_3_-MCI) and quantified the corresponding labelled urinary NMMA as main metabolite in individual urine samples collected over 48 h post dosage. The application of our analytical method on the quantification of M-12 to the previously collected urine samples might add some new details on human metabolism of MI and MCI. Thus, this study was aimed to investigate the human metabolism and renal excretion of M-12 after separate oral dosage of MI and MCI to volunteers to calculate metabolic conversion factors for M-12 for both compounds and basic pharmacokinetic parameters. To our knowledge, data on human metabolism of MI and/or MCI to the mercapturate M-12 have not been reported before.

## Experimental design

In January 2016, four healthy volunteers (2 m/2 f, age: 20–44 years, body weight: 75–100 kg) received an oral dose of 2 mg of either labelled ^13^C_3_-MI or D_3_-MCI (16.3 and 13 µMol, respectively) in 200 µL of ethanol in a glass of water separately and at least two weeks apart. An overview on the biometric and anamnestic data of the volunteers is given in Table 1.

**Table 1:** Anamnestic data of the human volunteers as described previously (Schettgen and Kraus, 2017)

	Volunteer 1	Volunteer 2	Volunteer 3	Volunteer 4
Sex	Male	Male	Female	Female
Age	44 years	21 years	20 years	23 years
Smoking status	Non-smoker	Non-smoker	Smoker	Non-smoker
Weight	94 kg	75 kg	100 kg	100 kg
Height	186 cm	186 cm	180 cm	172 cm
BMI	27.2	21.7	30.9	33.8

The resulting dosages of the isothiazolinones amounted to 20–26.7 µg/kg body weight and are three orders of magnitude lower than the lowest NOAEL from animal experiments. The volunteers provided urine samples before the dosage (*t*_0_) and consecutively collected their urine over the following 48 h. The time of the urine sample was noted by the volunteer. Urine volume for each void was determined by weighing difference of the filled and empty urine containers. The urine samples provided were aliquoted in 10-ml-aliquots and stored frozen at − 20 °C until analysis. Creatinine content of the urine samples was determined photometrically using the Jaffé-method (Larsen 1972).

### Chemicals

^13^C_3_-2-methylisothiazolinone and 5-chloro-2-(methyl-D_3_)-isothiazolinone was synthesized by Dr. Belov, Max Planck Institute for Biophysical Chemistry, Göttingen with a purity > 95% as determined by ^1^H-NMR and mass spectrometry. The 3-mercapturic acid conjugate of 3-thiomethyl-*N*-methyl-propionamide ((Acetylamino){[3-(methylamino)-1-(methylthio)-3-oxopropyl]thio}acetic acid) or shortly “M-12” was custom synthesized by the Institute of Organic and Biomolecular Chemistry (Göttingen, Germany) with an analytical purity > 98%. A deuterium-labelled internal standard of this metabolite (“D_3_-M-12”), namely (D_3_-Acetylamino){[3-(methylamino)-1-(methylthio)-3-oxopropyl]thio}acetic acid) was also custom synthesized by the Institute of Organic and Biomolecular Chemistry (Göttingen, Germany) with a purity of > 98% and an isotopic purity > 99%. The identification of the synthesis products was verified and proven by ^1^H-NMR spectra. The purity of the reference substances was controlled by HPLC–UV. All data for characterization of the standards are provided in the Supplemental Material to this manuscript.

Formic acid (100%) was supplied by Merck (Darmstadt, Germany). Acetonitrile (HPLC grade) and methanol (HPLC grade) were purchased from J.T. Baker (Germany). Ammonium formate was supplied by Fluka (Buchs, Suisse).

### Analytical procedure

The urinary excretion of labelled M-12 was quantified according to our previously published online SPE-LC/MS/MS-method (Schettgen et al 2021). 500 µL of urine were mixed with 500 µL of 100 mM ammonium formate buffer (pH 2.5). After addition of 10 µL formic acid and 10 µL of D_3_-M-12 (1 µg mL^−1^ in water) as internal standard, 100 µL of this solution are injected in the LC/MS/MS-system. The analyte is enriched and cleaned up from matrix interferences on a Phenomenex Strata-X-column (20 × 2 mm; 20 µm) using a mixture of water (pH 2.5) and methanol (90:10, v:v) and a flow rate of 0.5 ml/min. After 5 min, the analyte is backflushed on the analytical column [Phenomenex C18(2), 150 × 4.6 mm, 3 µm, 100 A] and separated from interferences using a gradient of water (pH 2.5) and acetonitrile. Tandem mass spectrometric determination was carried out on a Sciex API 5500 LC/MS/MS-system (Sciex, Darmstadt, Germany) in ESI-positive mode using the transitions 295.1 → 75.1 (Quantifier) and 295.1 → 100.1 (Qualifier1) and additionally 295.1 → 131.1 (Qualifier 2) for (unlabelled) M-12 as well as 298.1- > 75.1 for the internal standard and calibration. The different positions of the labelling (the internal standard is labelled in the *N*-acetyl moiety) allowed the quantification of ^13^C_3_-M-12 at the transition 298.1 → 78.0 and 298.1 → 104.0. Similarly, quantification of D_3_-labelled M-12 from the metabolism study of D_3_-MCI (labelled at the *N*-methyl moiety) was performed using the unique transition 298.1 → 134.1. The limit of quantification of the method was 0.2 µg/L urine for M-12. Urine samples with levels exceeding the upper linear range of 50 µg/L urine were diluted and re-analysed again. More details of the analytical method can be found in our recent publication (Schettgen et al. 2021). As previously described, the synthesised standards of M-12 and the internal standard D_3_-M-12 consisted of two diastereomers that were nearly separated in chromatography. It is interesting to note, that the investigated urine samples from the metabolism study mainly contained one of the two diastereomers. This has been observed in all urine samples investigated and corroborated our previous findings on the background excretion of M-12 in urine samples of the general population, where also one diastereomer was predominant. An exemplary chromatogram of the urine sample of a volunteer from the metabolism study of ^13^C_3_-MI is depicted in the Supplementary Files to this manuscript.

## Statistical analysis

The statistical analysis was performed using Microsoft Excel 2010. The decreasing metabolite levels after reaching the maximum excretion follow an exponential function with the following formula:

$$ c\left( t \right) = c_{{{\text{max}}}}  \times {\text{e}}^{{ - k\Delta t}} , $$

with *k* as urinary excretion constant, and Δ*t* as time after maximum concentration in hours. Thus, we calculated the individual urinary half-life for each volunteer and each dosage as natural logarithm of 2, divided by *k* (Fichtl 2001).

## Results and discussion

We obtained between 11 and 17 individual urine samples over 48 h with a total volume ranging from 3.91 to 6.39 L from the volunteers. We have applied our analytical method for the determination of labelled M-12 as metabolite of ^13^C_3_-MI and D_3_-MCI to all urine samples post dosage. While ^13^C_3_-M-12 could be quantified in all urine samples obtained after dosage in the 48-h time frame, D_3_-M-12 surprisingly could only be determined in the urine samples collected within 24 h after dosage.

**Fig. 2:**
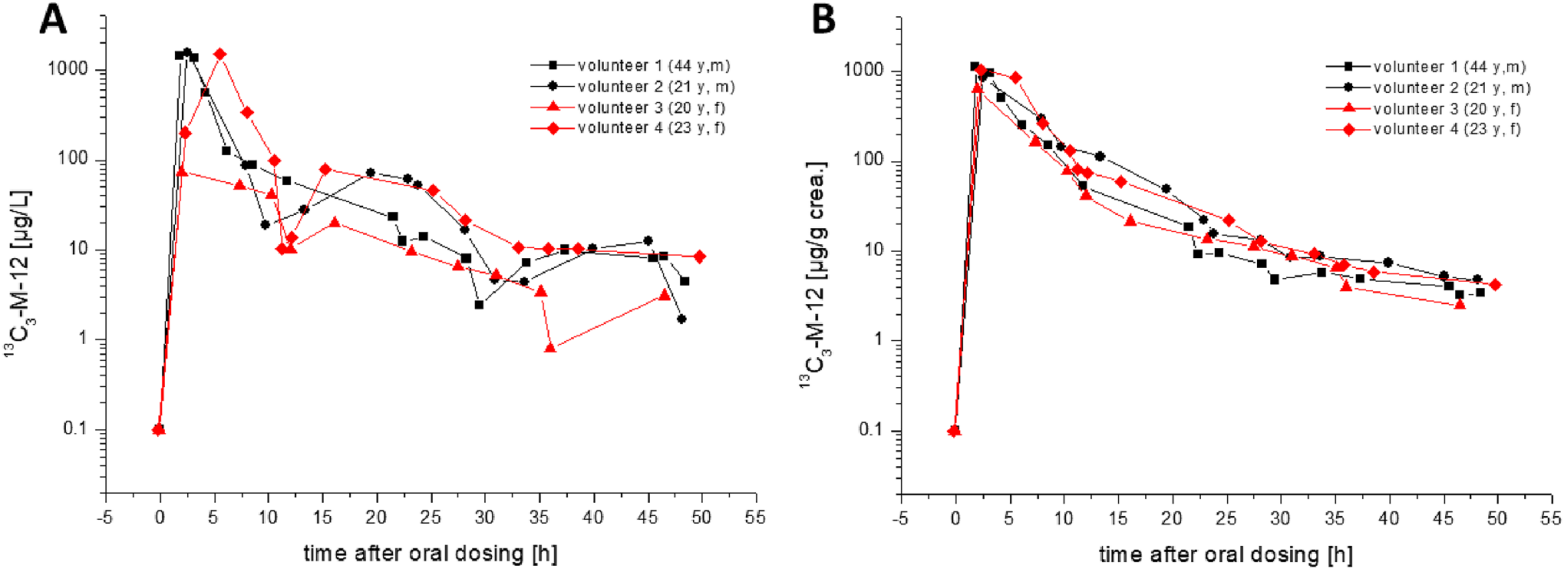
Urinary excretion kinetics of ^13^C_3_-M-12 after oral dosage of ^13^C_3_-MI in all volunteers (**A** volume-based levels, **B** creatinine-corrected levels)

**Fig. 3:**
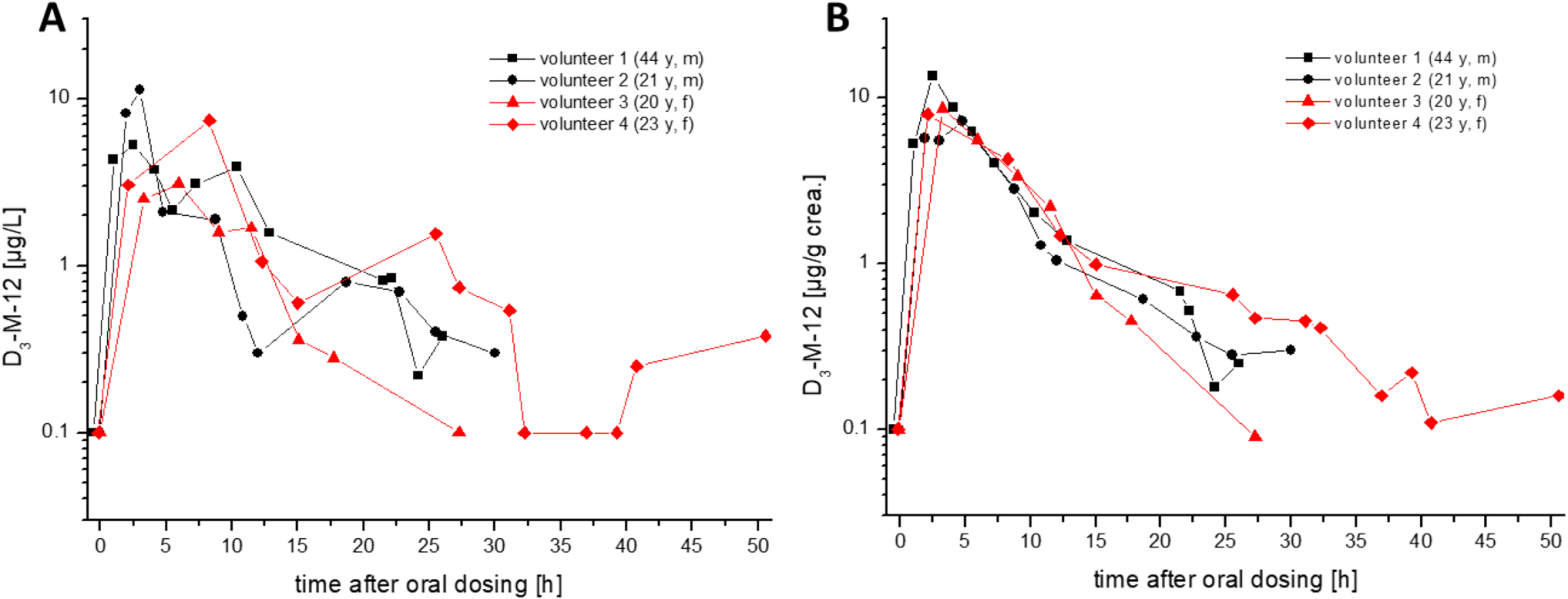
Urinary excretion kinetics of D_3_-M-12 after oral dosage of D_3_-MCI in all volunteers (**A** volume-based levels, **B** creatinine-corrected levels)

The urinary excretion kinetics of ^13^C_3_-M-12 after dosage of 2 mg ^13^C_3_-MI in all 4 volunteers are depicted in Fig. 2A, B on a logarithmic scale in µg/L and creatinine-adjusted values, respectively. Figure 3A, B show the respective kinetics of D_3_-M-12 after dosage of 2 mg D_3_-MCI in the same individuals.

**Fig. 4:**
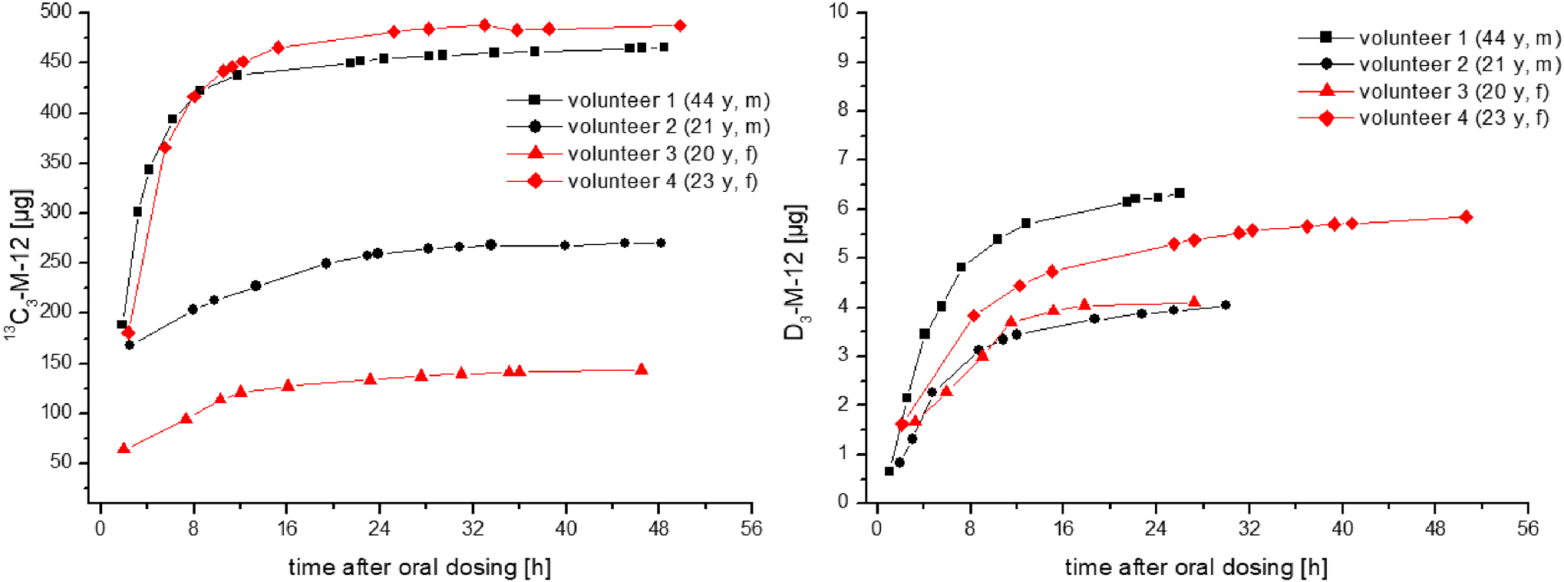
Cumulative excreted doses of ^13^C_3_-M-12 and D_3_-M-12 within 48 h post dosage of ^13^C_3_-MI and D_3_-MCI in all volunteers

The peak concentrations of ^13^C_3_-M-12 varied from 74 to 1588 µg/L (628–1110 µg/g creatinine) and were observed 2–3 h post dosage for ^13^C_3_-MI. In contrast, the maximum concentrations of D_3_-M-12 were orders of magnitude lower with levels ranging from 3.1 to 11.3 µg/L (7.2–13.6 µg/g creatinine) and the levels soon fell below the LOQ of our method. This was quite surprising and points to a clear difference in metabolism of MI and MCI, as the difference in quantitative excretion of the metabolite NMMA after dosing of MI and MCI was not so large (cf. Table 2). Thus, the mercapturate M-12 might be a suitable biomarker to differentiate between exposures to MI and MCI. This is most impressively shown in Fig. 4, where the cumulative urinary excretion of labelled M-12 is depicted both for oral exposure to MI (A) as well as MCI (B).

**Table 2:** Comparison of pharmacokinetic data for the mercapturate M-12 and the main metabolite NMMA calculated for all human volunteers and applied doses

Dosage		T_max_ [h]^*^		C_max_ [µg/g crea.]		T_½_ [h]^*^		F_UE_ (0 – 24 h)		F_UE_ (24 – 48 h)	
		NMMA	M-12	NMMA	M-12	NMMA	M-12	NMMA	M-12	NMMA	M-12
^13^C_3_-MI (16.2 µMol)	Volunteer 1	3.2	1.8	969	1110	5.0	3.3	30.0%	9.4%	0.9%	0.3%
	Volunteer 2	2.5	2.5	574	850	7.4	4.0	16.5%	5.4%	2.3%	0.2%
	Volunteer 3	2.0	2.0	951	628	6.5	2.8	16.4%	2.8%	1.6%	0.2%
	Volunteer 4	2.3	2.3	810	1032	5.4	4.2	26.4%	10.0%	0.8%	0.1%
	**Mean**	**2.5**	**2.2**	**826**	**905**	**6.1**	**3.6**	**22.3%**	**6.9%**	**1.4%**	**0.2%**
D_3_-MCI (13 µMol)	Volunteer 1	4.1	2.5	192	13.6	10.3	4.6	9.0%	0.16%	1.9%	–-
	Volunteer 2	1.9	4.8	288	7.2	6.5	5.0	12.0%	0.10%	0.8%	–-
	Volunteer 3	3.3	3.3	315	8.6	6.3	3.5	13.1%	0.11%	0.4%	–-
	Volunteer 4	2.1	2.1	333	8.0	7.4	7.0	14.5%	0.14%	1.4%	–-
	**Mean**	**2.9**	**3.2**	**282**	**9.4**	**7.6**	**5.0**	**12.2%**	**0.13%**	**1.1%**	**–-**

^*^calculated using creatinine-corrected values

F_UE:_ urinary excretion factor

The elimination curves for M-12 were quite similar for all four volunteers, both for dosage of ^13^C_3_-MI as well as for D_3_-MCI. It seems that there is no striking difference in kinetics of M-12 between male and female volunteers, similar as previously reported for the main metabolite NMMA. As clearly visible in Figs. 2 and 3, creatinine correction considerably smoothed the elimination curves. We have summarized the whole dataset for all volunteers and all dosages both for excretion of M-12 as well as NMMA as main metabolite in the supplemental information to this article (Supplement Table 1 + 2). The renal clearance of M-12 for all dosages and volunteers is also displayed in the Supplement (Figure S2).

The excretion of M-12 was even quicker than previously shown for NMMA, showing maximum concentrations usually in the first urine sample collected post dosage. Excretion of M-12 occurred via first order kinetics for both dosages. The urinary half-life for M-12 was calculated as described under “Statistical analysis” using the creatinine-corrected excretions. The individual half-lifes ranged from 2.8 to 4.2 h (mean: 3.6 h) after dosage of ^13^C_3_-MI and a slightly slower half-life ranging from 3.0 to 7.0 h (mean: 5.0 h) after dosage of D_3_-MCI. Consequently, excretion of M-12 was almost complete within 24 h post dosage of MI with only 0.2% excreted from 24 to 48 h. For MCI, there was hardly any excretion of M-12 detectable after 24 h. Under consideration of the molar masses, the mean total excreted amount of labelled M-12 (urinary excretion factor, *F*_UE_) within 48 h post dosage amounts to 7.1% (range 3.0–10.1%) after dosage of MI and only 0.13% (range 0.10–0.16%) after dosage of MCI. In animal experiments on Sprague–Dawley rats, a slightly higher urinary excretion of 10–23% of the dose was reported for the mercapturate M-12 after oral dosage of radiolabeled MI (Burnett et al. 2010). This difference is probably due to the known higher activity of glutathione-S-transferase in rat vs. human liver (Baars et al. 1981). For MCI, we found no quantitative data on animal metabolism to M-12 in literature.

In summary, the mercapturate M-12 can be regarded as a second major human metabolite of MI, but only as a minor human metabolite of MCI according to our results. We might speculate that the chlorine atom of MCI is directly attacked by glutathione before ring opening within human metabolism, leading to a different glutathione conjugate and thus another metabolic pattern compared to the unsubstituted MI. In Table 2 we have compared the pharmacokinetic data obtained for all volunteers and both dosages both for the previously reported main metabolite NMMA as well as for the mercapturate M-12.

The fact that urinary excretion of M-12 is specific for exposure to MI opens up the possibility for a differential exposure estimation based on urinary excretion of M-12 and NMMA, for example in population studies. Exclusive exposure to MI can be calculated based on the urinary F_UE_ for M-12 determined in this study. Comparison with exposure estimates for MCI/MI (3:1) based on the concentration of urinary NMMA and the respective F_UEs_ determined in our previous investigation might give some upper and lower boundaries for the different exposure sources and potentially point to different contributions.

## Conclusion

With this study we complement the picture about human metabolism of the biocidal active substances MI and MCI with the quantification of the urinary mercapturate M-12. As we used isotopically labelled substances for the oral dosing, we could unequivocally quantify urinary labelled M-12 without the influence of individual background levels. M-12 has previously been reported as a major metabolite of MI in rats. Our derived human metabolic conversion factors of 7.1% for MI confirm that M-12 is also a major human metabolite. However, M-12 turned out to be only a minor metabolite of MCI with a metabolic conversion factor of 0.13%. Thus, the quantification of M-12 in urine samples of the general population is highly specific for individual exposures to MI. In consequence, the parallel quantification of NMMA as common metabolite of MCI and MI together with the quantification of the mercapturate M-12 might allow to discriminate between exposures to the standalone biocide MI and the mixture of MCI/MI (3:1). Thus, our study provides the basis for a differential exposure estimation and risk assessment of both biocides in population studies.

## Supplementary Information

Below is the link to the electronic supplementary material.

Supplementary file1 (DOCX 190 kb)

Supplementary file2 (DOCX 3921 kb)
