# Supplementary material for: New data on the metabolism of chloromethylisothiazolinone and methylisothiazolinone in human volunteers after oral dosage: excretion kinetics of a urinary mercapturic acid metabolite (“M-12”)
Source: Arch Toxicol. 2021 Jun 21;95(8):2659–65. doi: 10.1007/s00204-021-03100-5 (PMC8298359; doi:10.1007/s00204-021-03100-5)

Supplemental Table 1: Excretion of urinary ^13^C_3_-NMMA and ^13^C_3_-M-12 after oral dosage of 2 mg of ^13^C_3_-MI (16.2 µMol) in four human volunteers.

| Volunteer 1 (m) | | | | Volunteer 2 (m) | | | | Volunteer 3 (f) | | | | Volunteer 4 (f) | | | |
| --- | --- | --- | --- | --- | --- | --- | --- | --- | --- | --- | --- | --- | --- | --- | --- |
| time  [h] | crea.  [g/L] | NMMA  [µg/L] | M-12  [µg/L] | time  [h] | crea.  [g/L] | NMMA  [µg/L] | M-12  [µg/L] | time  [h] | crea.  [g/L] | NMMA  [µg/L] | M-12  [µg/L] | time  [h] | crea.  [g/L] | NMMA  [µg/L] | M-12  [µg/L] |
| 0 | 1.74 | < 0.5 | < 0.2 | 0 | 1.63 | < 0.5 | < 0.2 | 0 | 0.81 | < 0.5 | < 0.2 | 0 | 0.64 | < 0.5 |  |
| 1.83 | 1.30 | 1194.9 | 1445 | 2.50 | 1.87 | 1073.3 | 1588 | 2.00 | 0.12 | 111.8 | 74 | 2.33 | 0.19 | 158.0 | 201 |
| 3.17 | 1.42 | 1375.4 | 1341 | 7.92 | 0.29 | 112.8 | 86 | 7.33 | 0.32 | 174.4 | 52 | 5.50 | 1.77 | 1073.2 | 1513 |
| 4.17 | 1.10 | 763.1 | 556 | 9.75 | 0.13 | 29.5 | 18.8 | 10.25 | 0.52 | 106.6 | 41 | 8.00 | 1.28 | 603.3 | 337 |
| 6.17 | 0.49 | 187.5 | 125 | 13.33 | 0.24 | 58.5 | 27.5 | 12.00 | 0.25 | 25.8 | 10.1 | 10.50 | 0.75 | 229.9 | 99 |
| 8.50 | 0.58 | 162.4 | 88 | 19.42 | 1.50 | 235.5 | 73 | 16.08 | 0.94 | 89.6 | 20.1 | 11.25 | 0.13 | 22.5 | 10.2 |
| 11.75 | 1.09 | 209.3 | 58 | 22.92 | 2.73 | 261.2 | 61 | 23.17 | 0.71 | 37.8 | 9.6 | 12.17 | 0.19 | 32.4 | 13.8 |
| 21.50 | 1.24 | 92.1 | 23.0 | 23.83 | 3.38 | 202.3 | 52 | 27.50 | 0.59 | 20.5 | 6.5 | 15.25 | 1.31 | 176.1 | 79 |
| 22.33 | 1.35 | 83.9 | 12.5 | 28.17 | 1.24 | 61.2 | 16.4 | 31.00 | 0.59 | 17.6 | 5.2 | 25.17 | 2.12 | 106.2 | 46.3 |
| 24.33 | 1.46 | 47.6 | 13.9 | 30.83 | 0.54 | 19.2 | 4.6 | 35.08 | 0.51 | 11.3 | 3.4 | 28.17 | 1.69 | 34.6 | 21.5 |
| 28.25 | 1.11 | 23.3 | 8.0 | 33.58 | 0.51 | 13.9 | 4.4 | 36.00 | 0.19 | 2.6 | 0.8 | 33.00 | 1.16 | 13.1 | 10.8 |
| 29.42 | 0.50 | 4.7 | 2.4 | 39.92 | 1.40 | 25.4 | 10.3 | 46.50 | 1.23 | 7.4 | 3.1 | 35.75 | 1.45 | 10.8 | 10.2 |
| 33.83 | 1.24 | 9.3 | 7.2 | 45.08 | 2.34 | 27.6 | 12.3 |  |  |  |  | 38.50 | 1.74 | 9.6 | 10.2 |
| 37.33 | 2.04 | 10.8 | 10.0 | 48.17 | 0.35 | 2.8 | 1.7 |  |  |  |  | 49.75 | 1.97 | 6.0 | 8.4 |
| 45.50 | 2.05 | 5.3 | 8.2 |  |  |  |  |  |  |  |  |  |  |  |  |
| 46.50 | 2.68 | 4.5 | 8.5 |  |  |  |  |  |  |  |  |  |  |  |  |
| 48.42 | 1.28 | 2.2 | 4.4 |  |  |  |  |  |  |  |  |  |  |  |  |
|  |  |  |  |  |  |  |  |  |  |  |  |  |  |  |  |
| Total excreted volume: 3900 mL  Total excreted dose  NMMA: 601 µg = 30.9 %  M-12: 466 µg = 9.7 % | | | | Total excreted volume: 4000 mL  Total excreted dose  NMMA: 365 µg = 18.8 %  M-12: 270 µg = 5.6 % | | | | Total excreted volume: 6400 mL  Total excreted dose:  NMMA: 351 µg = 18.0 %  M-12: 144 µg = 3.0 % | | | | Total excreted volume: 4010 mL  Total excreted dose  NMMA: 528 µg = 27.2 %  M-12: 488 µg = 10.1 % | | | |

Supplemental Table 2: Excretion of urinary D_3_-NMMA after oral dosage of 2 mg of D_3_-MCI (13.0 µMol) in four human volunteers.

| Volunteer 1 (m) | | | | Volunteer 2 (m) | | | | Volunteer 3 (f) | | | | Volunteer 4 (f) | | | |
| --- | --- | --- | --- | --- | --- | --- | --- | --- | --- | --- | --- | --- | --- | --- | --- |
| time  [h] | crea.  [g/L] | NMMA  [µg/L] | M-12  [µg/L] | time  [h] | crea.  [g/L] | NMMA  [µg/L] | M-12  [µg/L] | time  [h] | crea.  [g/L] | NMMA  [µg/L] | M-12  [µg/L] | time  [h] | crea.  [g/L] | NMMA  [µg/L] | M-12  [µg/L] |
| 0 | 2.43 | < 0.5 | < 0.2 | 0 | 2.70 | < 0.5 | < 0.2 | 0 | 1.29 | < 0.5 | < 0.2 | 0 | 2.03 | < 0.5 | < 0.2 |
| 1.00 | 0.82 | 114.7 | 4.4 | 1.92 | 1.43 | 412.4 | 8.2 | 3.25 | 0.29 | 91.8 | 2.5 | 2.08 | 0.38 | 127.5 | 3.1 |
| 2.50 | 0.39 | 57.8 | 5.3 | 3.00 | 2.03 | 579.2 | 11.3 | 5.92 | 0.55 | 127.1 | 3.1 | 8.25 | 1.75 | 288.4 | 7.5 |
| 4.08 | 0.43 | 82.1 | 3.8 | 4.75 | 0.29 | 64.0 | 2.1 | 9.00 | 0.47 | 79.1 | 1.6 | 12.25 | 0.72 | 44.1 | 1.1 |
| 5.50 | 0.34 | 28.7 | 2.2 | 8.75 | 0.67 | 95.1 | 1.9 | 11.50 | 0.77 | 67.9 | 1.7 | 15.00 | 0.60 | 28.3 | 0.6 |
| 7.25 | 0.77 | 48.9 | 3.1 | 10.83 | 0.41 | 33.4 | 0.5 | 15.08 | 0.57 | 20.5 | 0.4 | 25.50 | 2.42 | 94.3 | 1.6 |
| 10.33 | 1.92 | 110.7 | 3.9 | 12.00 | 0.28 | 21.6 | 0.3 | 17.75 | 0.63 | 22.1 | 0.3 | 27.25 | 1.57 | 57.2 | 0.7 |
| 12.83 | 1.14 | 57.6 | 1.6 | 18.67 | 1.35 | 77.5 | 0.8 | 27.25 | 1.14 | 29.2 | < 0.2 | 31.08 | 1.20 | 22.7 | 0.5 |
| 21.50 | 1.21 | 40.3 | 0.8 | 22.75 | 2.03 | 65.3 | 0.7 | 31.08 | 0.25 | 2.4 | < 0.2 | 32.25 | 0.25 | 3.1 | < 0.2 |
| 22.17 | 1.61 | 49.6 | 0.8 | 25.50 | 1.43 | 32.5 | 0.4 | 31.58 | 0.13 | 1.0 | < 0.2 | 36.92 | 0.70 | 6.6 | < 0.2 |
| 24.17 | 1.22 | 36.1 | 0.2 | 30.00 | 1.10 | 15.1 | 0.3 | 33.25 | 0.28 | 1.9 | < 0.2 | 39.25 | 0.68 | 3.8 | < 0.2 |
| 26.00 | 1.52 | 40.3 | 0.4 | 31.42 | 0.30 | 2.3 | < 0.2 | 35.75 | 0.44 | 2.7 | < 0.2 | 40.75 | 2.33 | 12.0 | 0.2 |
| 30.00 | 1.38 | 18.1 | < 0.2 | 31.83 | 0.24 | 1.3 | < 0.2 | 43.42 | 0.55 | 1.8 | < 0.2 | 50.58 | 2.28 | 8.3 | 0.4 |
| 36.83 | 2.18 | 22.4 | < 0.2 | 34.17 | 1.50 | 10.8 | < 0.2 | 47.25 | 0.82 | 2.2 |  |  |  |  |  |
| 45.50 | 1.57 | 13.5 | < 0.2 | 42.25 | 1.46 | 6.9 | < 0.2 |  |  |  |  |  |  |  |  |
| 48.00 | 1.18 | 8.0 | < 0.2 | 46.75 | 2.32 | 8.6 | < 0.2 |  |  |  |  |  |  |  |  |
|  |  |  |  |  |  |  |  |  |  |  |  |  |  |  |  |
| Total excreted volume: 4030 mL  Total excreted dose  NMMA: 171 µg = 10.9 %  M-12: 6.3 µg = 0.16 % | | | | Total excreted volume: 4300 mL  Total excreted dose  NMMA: 216 µg = 13.8 %  M-12: 4.1 µg = 0.10 % | | | | Total excreted volume: 6350 mL  Total excreted dose  NMMA: 196 µg = 12.5 %  M-12: 4.1 µg = 0.11 % | | | | Total excreted volume: 4590 mL  Total excreted dose  NMMA: 249 µg = 15.9 %  M-12: 5.8 µg = 0.15 % | | | |

Supplemental Figure S2: Renal clearance (µg/h) of M-12 after oral dosage of 2 mg of ^13^C_3_-MI (16.2 µMol) (A) or D_3_-MCI (13.0 µMol) in four human volunteers.


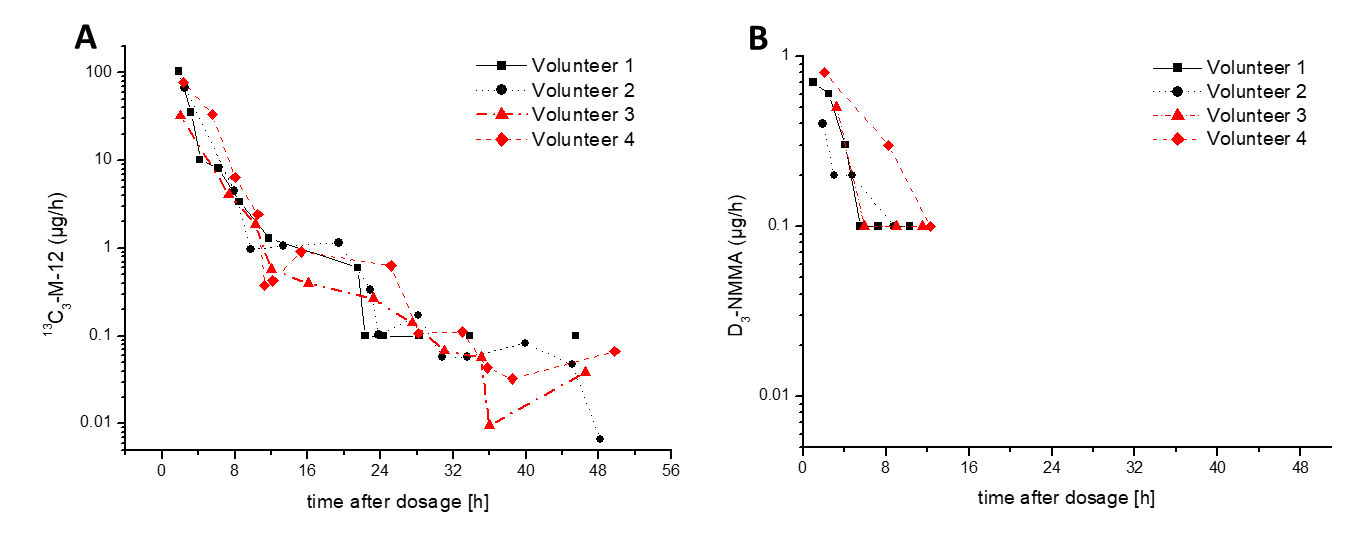


Supplemental File 1: ^1^H-NMR spectrum of synthesized M-12


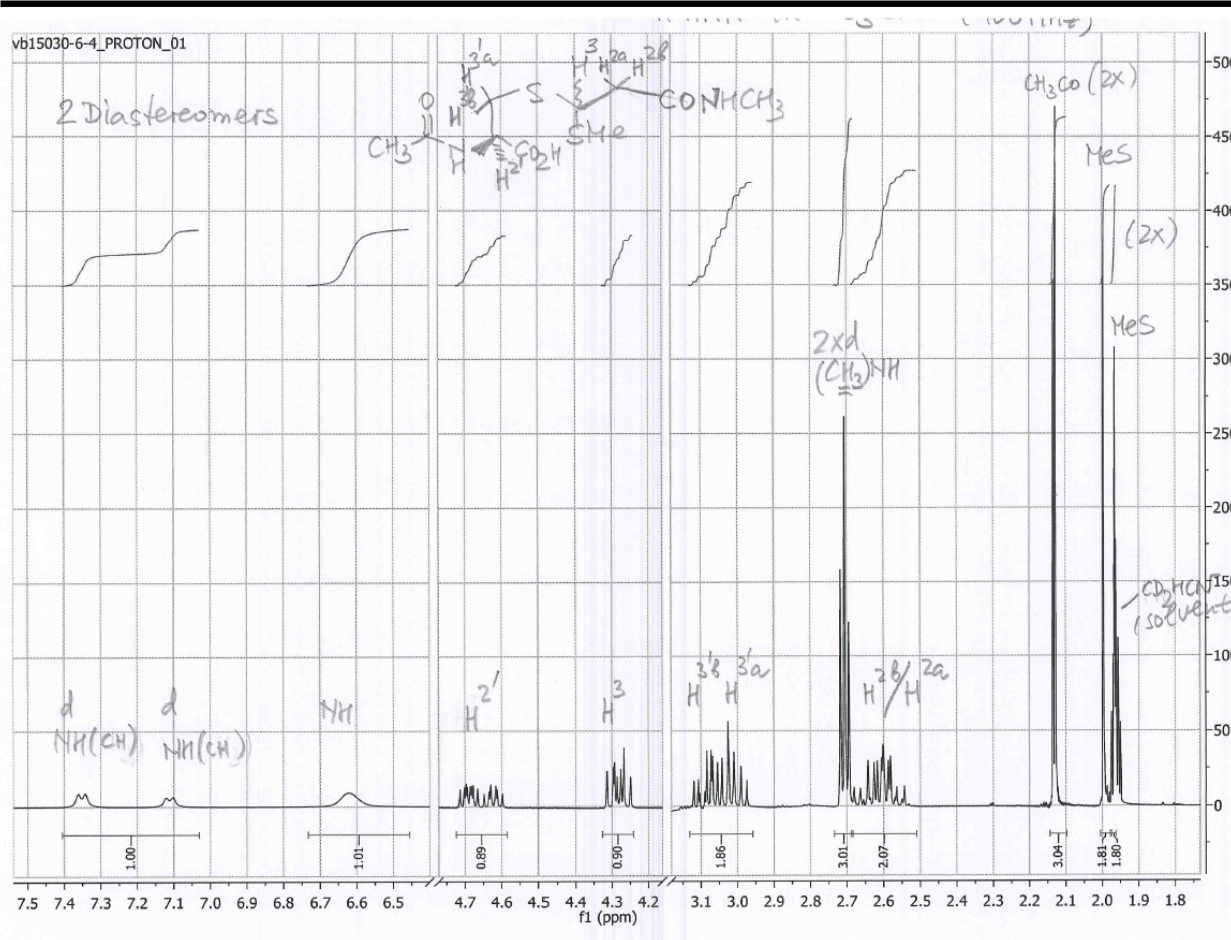


Supplemental File 2: HPLC-UV-chromatogram of synthesized M-12


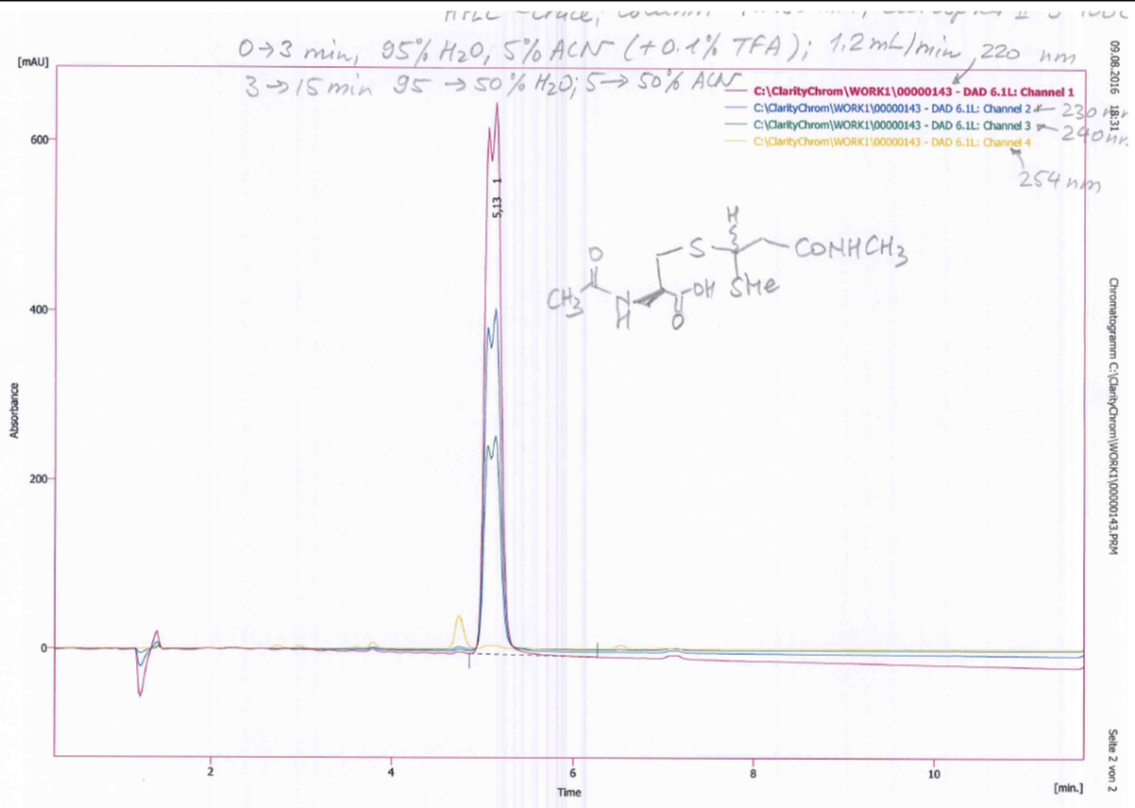


Supplemental File 3: ^1^H-NMR spectrum of synthesized D3-M-12


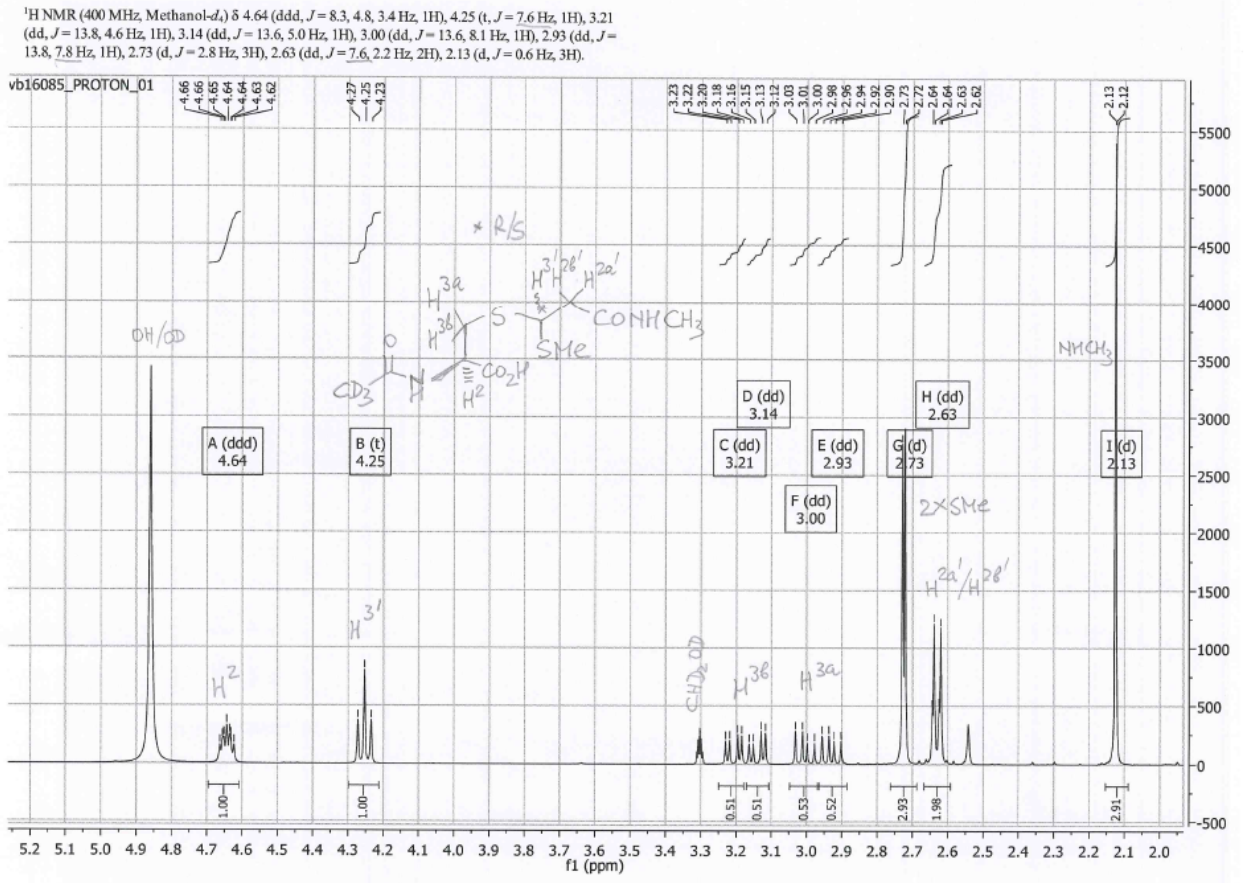


Supplemental File 4: HPLC-UV-chromatogram of synthesized D3-M-12


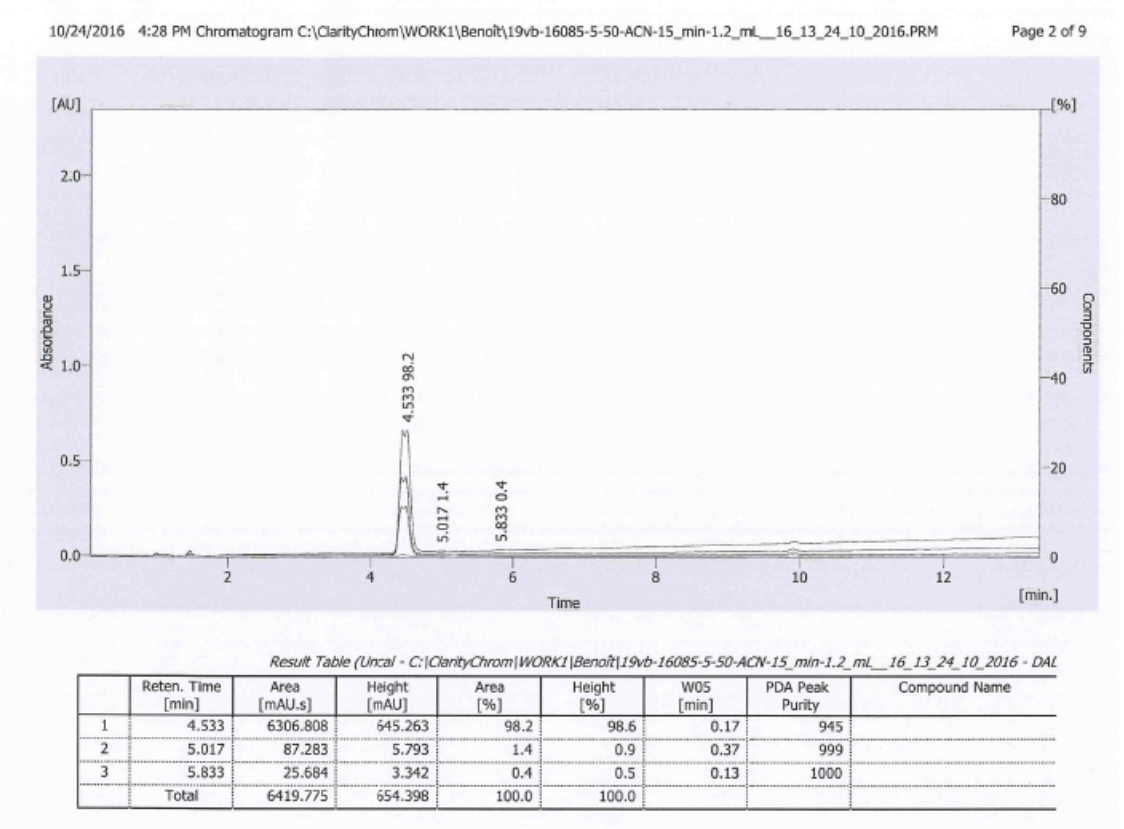


Supplemental File 5: ^1^H-NMR of synthesized ^13^C_3_-MI.

Supplemental File 6: EI mass spectrum of synthesized ^13^C_3_-MI.


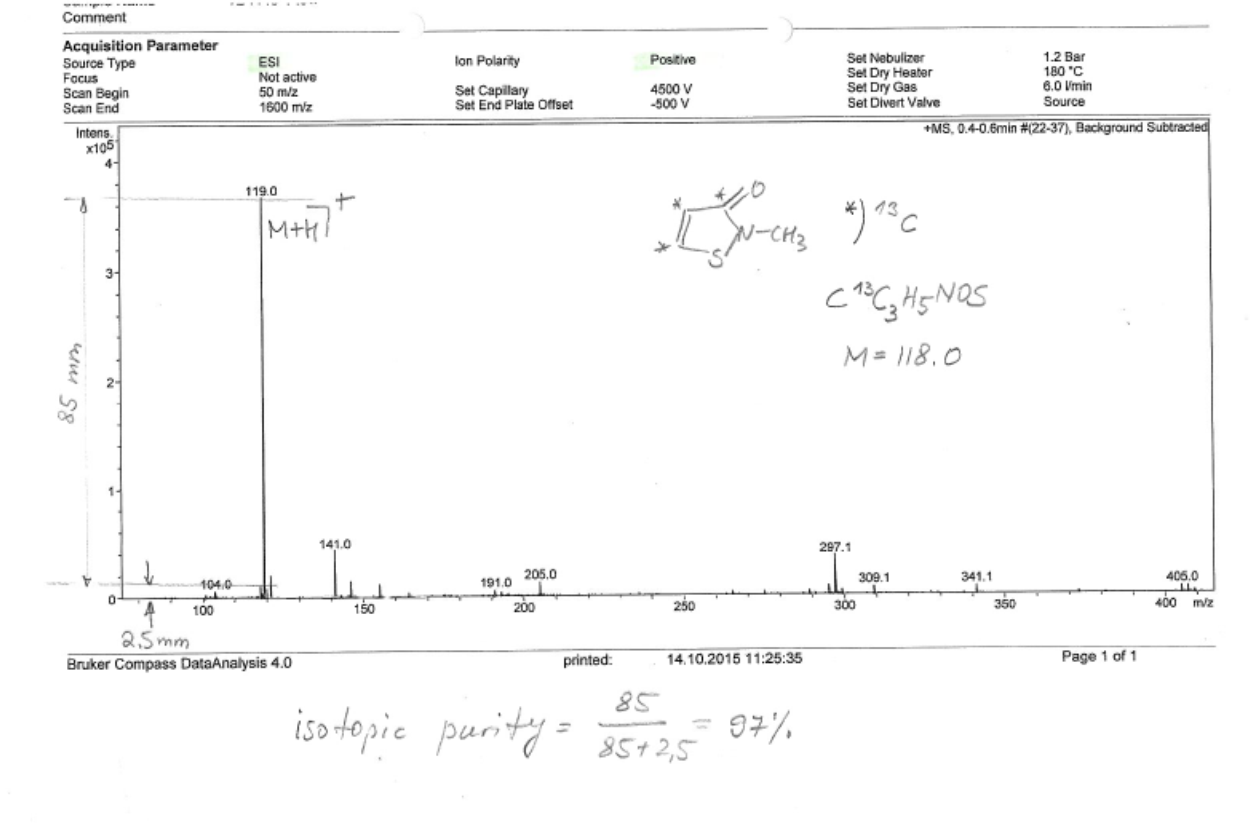


Supplemental File 7: GC/EI-MS-spectrum of synthesized D_3_-MCI.


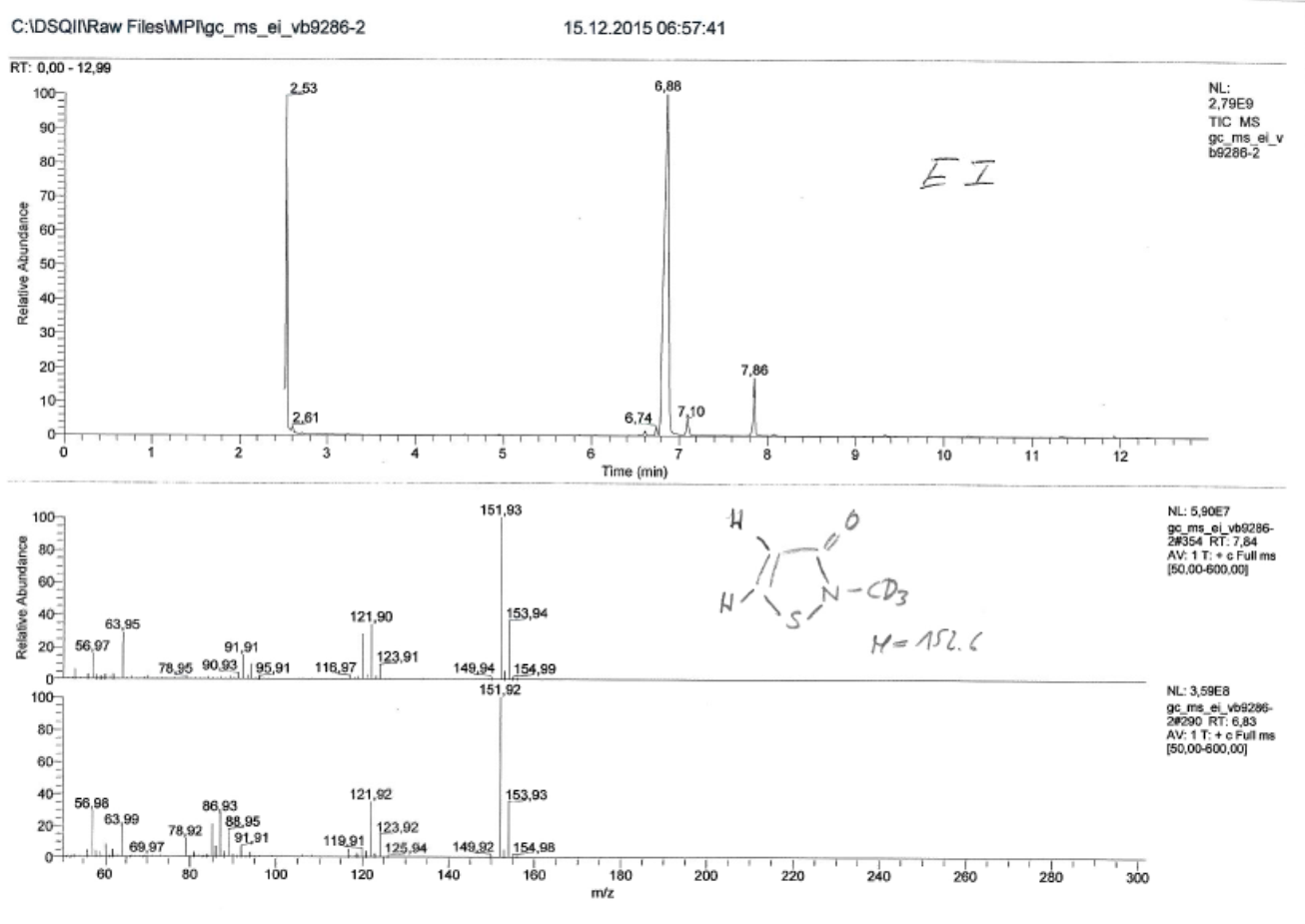

Supplement: Supplementary file 1 — Supplementary file1 (DOCX 190 kb) [file 204_2021_3100_MOESM1_ESM.docx]
