# Supplementary material for: New data on the metabolism of chloromethylisothiazolinone and methylisothiazolinone in human volunteers after oral dosage: excretion kinetics of a urinary mercapturic acid metabolite (“M-12”)
Source: Arch Toxicol. 2021 Jun 21;95(8):2659–65. doi: 10.1007/s00204-021-03100-5 (PMC8298359; doi:10.1007/s00204-021-03100-5)

Supplemental Figure S1: Exemplary chromatogram of the processed urine sample of a volunteer (44 yrs, m) obtained after oral dosing of ^13^C_3_-MI with a level of ^13^C_3_-M-12 of 88 µg/L (crea.: 0.58 g/L).


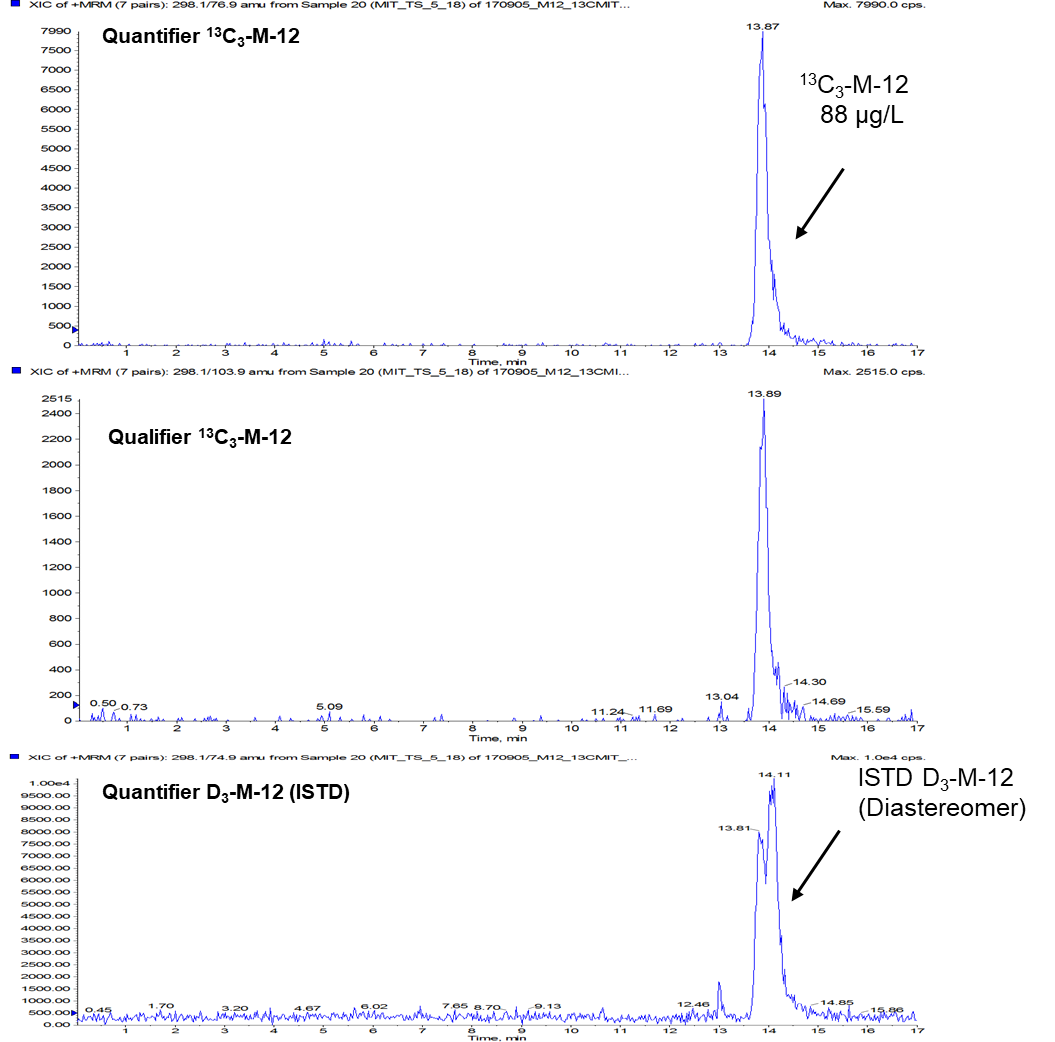

Supplement: Supplementary file 2 — Supplementary file2 (DOCX 3921 kb) [file 204_2021_3100_MOESM2_ESM.docx]
